# High-Mobility Group Box 1 expression predicts survival of patients after resection of adenocarcinoma of the ampulla of Vater

**DOI:** 10.1186/s12957-019-1675-8

**Published:** 2019-08-09

**Authors:** Takashi Murakami, Ryusei Matsuyama, Michio Ueda, Yasuhisa Mochizuki, Yuki Homma, Kunio Kameda, Keiichi Yazawa, Yusuke Izumisawa, Tadao Fukushima, Nobuyuki Kamimukai, Kenichi Yoshida, Noriyuki Kamiya, Robert M. Hoffman, Itaru Endo

**Affiliations:** 1 Department of Gastroenterological Surgery, Graduate School of Medicine, Yokohama City University, 3-9, Fukuura, Kanazawa-ku, Yokohama, 236-0004 Japan; 2 Department of Surgery, Gastroenterological Center, Yokohama City University, Yokohama, Japan; 3 Department of Gastroenterological Surgery, Yokohama Municipal Citizen’s Hospital, Yokohama, Japan; 4 Department of Surgery, Yokosuka Kyosai Hospital, Yokosuka, Japan; 5 Department of Surgery, Yokosuka City Hospital, Yokosuka, Japan; 6 Department of Surgery, Yokohama City Minato Red Cross Hospital, Yokohama, Japan; 7 Department of Gastroenterological Surgery, Fujisawa City Hospital, Fujisawa, Japan; 8 Department of Surgery, Saiseikai Yokohama Nanbu Hospital, Yokohama, Japan; 9 Department of Surgery, Yokohama Hodogaya Central Hospital, Yokohama, Japan; 10 Department of Surgery, Saiseikai Wakakusa Hospital, Yokohama, Japan; 11 Department of Surgery, Ito Municipal Hospital, Ito, Japan; 12 Department of Surgery, University of California, San Diego, California USA; 13 AntiCancer, Inc., San Diego, California USA

**Keywords:** HMGB1, Adenocarcinoma of the ampulla of Vater, Overall survival

## Abstract

**Background:**

Expression of High-Mobility Group Box 1 (HMGB1), a multifunctional protein involved in DNA function as well as cell proliferation, inflammation, and the immune response, has been reported to be prognostic in several types of malignancies. However, the prognostic value of HMGB1 in ampullary cancer has not been studied.

**Methods:**

Patients with adenocarcinoma of the ampulla of Vater who underwent R0 resection with pancreaticoduodenectomy between 2001 and 2011 were included in the present multi-institutional study. The degree of HMGB1 expression was examined in each resected specimen by immunohistochemical staining.

**Results:**

A total of 101 patients were enrolled of which, 79 patients were eligible. High expression of HMGB1 was observed in 31 (39%) patients. Blood loss, transfusion, tumor stage, nodal status, and HMGB1 expression were identified as predictors with univariate analysis. Multivariate analysis showed that transfusion, lymph-node metastasis, and high HMGB1 expression were independent predictors of poor overall survival. Subgroup analysis showed that high HMGB1 expression was predictive, especially in patients who did not receive adjuvant chemotherapy.

**Conclusions:**

High HMGB1 expression is an independent predictor of poor prognosis in patients with adenocarcinoma of the ampulla of Vater not treated with adjuvant chemotherapy.

## Introduction

Ampullary cancer is a rare biliary tract cancer that accounts for 0.2% of digestive tract cancers in the USA [1]. The Japanese Society of Hepato-Biliary-Pancreatic Surgery reported a total of 13,192 bile duct cancer cases, including 2161 (16.4%) ampullary cancer patients in 2016 [2]. Ampullary cancer patients are usually diagnosed at an early stage, with 30% of tumors localized in the mucosa or the sphincter of Oddi [2]. Therefore, more than half of the patients are treated with surgical resection, achieving relatively better long-term outcomes than other biliary tract cancers or pancreatic cancer [2, 3]. However, considering that lymph-node metastasis occurs even at an early stage of ampullary cancer, and the 5-year survival for patients with lymph-node metastasis is less than 30%, improvement of patient outcome is still needed [2, 4]. Discovery of new predictive markers can provide new therapeutic targets that could result in further improvements in long-term outcome.

Expression of High-Mobility Group Box 1 (HMGB1), a multifunctional protein involved in DNA function as well as other cell functions, has been shown to be a prognostic factor in many cancers, such as esophageal cancer, gastric cancer, colorectal cancer, pancreatic cancer, and hepatocellular carcinoma [5]. However, the relationship between HMGB1 overexpression and survival in ampullary cancer has not yet been elucidated. Therefore, the present study was performed to determine the prognostic impact of HMGB1 in adenocarcinoma of the ampulla of Vater. Because of the rarity of the disease, clinicopathological data and pathological specimens from ampullary cancer patients were collected through a multi-institutional collaboration.

## Patients and methods

### YCOG 1205, a multi-institutional study

The Yokohama Clinical Oncology Group (YCOG) is a study group for investigating various surgical and oncological issues. The present multi-institutional, retrospective study was conducted as YCOG 1205. Eleven institutions including the Department of Gastroenterological Surgery, Graduate School of Medicine, Yokohama City University; the Department of Surgery, Gastroenterological Center, Yokohama City University; the Department of Gastroenterological Surgery, Yokohama Municipal Citizen’s Hospital; the Department of Surgery, Yokosuka Kyosai Hospital; the Department of Surgery, Yokosuka City Hospital; the Department of Surgery, Yokohama City Minato Red Cross Hospital; the Department of Gastroenterological Surgery, Fujisawa City Hospital; the Department of Surgery, Saiseikai Yokohama Nanbu Hospital; the Department of Surgery, Yokohama Hodogaya Central Hospital; the Department of Surgery, Saiseikai Wakakusa Hospital; and the Department of Surgery, Ito Municipal Hospital were involved. The study was conducted according to the Helsinki Declaration. The main institution, the Department of Gastroenterological Surgery, Graduate School of Medicine, Yokohama City University (IRB approval number: B120705037), and all other participating institutions approved the study protocol.

### Inclusion criteria

Ampullary cancer was defined as a cancer derived from tissue surrounded by the Oddi muscle [6]. Patients with ampullary cancer treated with pancreaticoduodenectomy in YCOG were enrolled in the present study. Patients who met all of the following criteria were eligible for the present study: adenocarcinoma of the ampulla of Vater confirmed by histological examination; achievement of R0 resection by pancreaticoduodenectomy (PD), pylorus-preserving PD (PpPD), or subtotal stomach-preserving PD (SSPPD) between 2001 and 2011; and availability of tissue specimens. Pathologists in each institute reviewed the histological analysis. Clinicopathological data and paraffin-embedded resected tissue specimens or unstained slides from each participant were obtained and collected in the Department of Gastroenterological Surgery, Graduate School of Medicine, Yokohama City University.

### Definition of lymph-node dissection for ampullary cancer

The level of lymph-node dissection for ampullary cancer was classified into the following three categories according to the General Rules for Surgical and Pathological Studies on Cancer of the Biliary Tract, 5th edition [6]: D1, dissection of retro-pancreatic lymph-nodes; D2, dissection of pre-pancreatic lymph-nodes, mesenteric lymph-nodes (proximal portion of the superior mesenteric artery, inferior pancreaticoduodenal artery, middle colic artery, and jejunal artery), and lymph-nodes along the distal bile duct in addition to D1 dissection; and D3, dissection of para-aortic lymph-nodes and lymph-nodes around the celiac trunk, common hepatic artery, and hepato-duodenal ligament along with D2 dissection.

### Immunohistochemical staining

Fresh tumor samples were fixed in formalin and embedded in paraffin before sectioning and staining. Tissue sections (4-μm-thick) from the center of the tumors were deparaffinized in xylene and rehydrated in an ethanol series. Antigen retrieval was achieved by autoclaving in a citrate buffer (pH 6.0). Intrinsic peroxidase activity was quenched in a hydrogen peroxide phosphate-buffered saline for 30 min at room temperature. Then, bovine serum albumin (10%) was added to block non-specific antibody binding. After blocking non-specific antibody binding, sections were incubated with the primary antibody, rabbit polyclonal anti-HMGB1 antibody (ab18256, Abcam, Cambridge, UK), at 4 °C overnight. The bound primary antibody was detected with an anti-rabbit secondary antibody. The labeled antigens were visualized with DAB. Sections were counterstained with hematoxylin.

### HMGB1 expression analysis

**Fig. 1:**
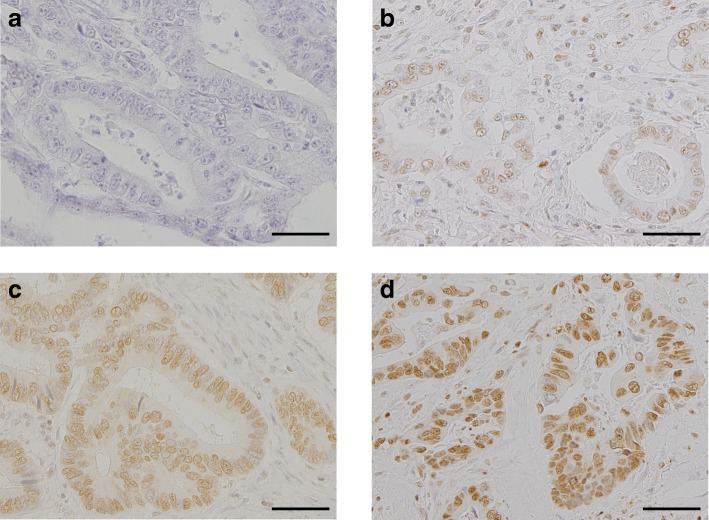
Immunohistochemical staining images of HMGB1 expression in adenocarcinoma of the ampulla of Vater. Examples of HMGB1 expression in the nucleus of cancer cells. **a** No staining (intensity = 1). **b** Light brown staining (intensity = 2). **c** Moderate brown staining (intensity = 3). **d** Dark brown staining (intensity = 4). Scale bars = 100 μm

The expression level of HMGB1 was evaluated by nuclear-staining intensity according to previously reported methods [7]. Four categories of staining were used: 1, no stain, 2–4, increasing intensity of brown stain (Fig. 1). Four categories of positivity were used, each with an increase of 25%. Positivity scores were multiplied to obtain a histochemical score. High expression was defined as a score of greater than or equal to 8, and low expression was defined as no more than 6.

### Statistical analysis

Statistical analyses were with SPSS statistics version 21.0 (IBM, New York City, NY, USA). The chi-squared test was used for statistical significance of categorical variables. The Mann-Whitney *U* test was used for continuous variables. Kaplan-Meier analysis was used for survival and compared by the log-rank test. The Cox proportional hazards model was used for multivariate analysis on the outcome. Significant variables on univariate analysis were included in the multivariate analysis. A *P* value < 0.05 was considered significant.

## Results

### Patient characteristics

A total of 101 patients were enrolled, of which 79 patients were eligible for the present study using the criteria described above under the “Patients and methods” section. The patients’ characteristics are summarized in Table 1. High expression of HMGB1 was confirmed in 31 (39%) patients. Forty-five patients underwent conventional PD, and 31 underwent PpPD, while 3 patients were treated with SSPPD. Lymph-node dissection greater than D2 was performed on 55 (70%) patients. Median operation time and blood loss were 466 min and 850 ml, respectively; 24% of patients were transfused. Morbidity of greater than grade IIIa, according to the Clavien-Dindo classification, occurred in 35 (44%) patients [8]. Of these cases, 20 had a grade B or C postoperative pancreatic fistula, defined by the International Study Group of Pancreatic Fistula classification [9]. According to the 8th Edition of the American Joint Committee on Cancer Staging Manual, there were 20 patients in stage IA, 18 in stage IB, 15 in stage IIA, 22 in stage IIIA, and 4 in stage IIIB [10]. Gemcitabine monotherapy was administered to 19 patients, and S-1 monotherapy was given to 7 patients. Three patients were treated with UFT, and intra-arterial chemotherapy using a 5-FU plus cisplatin regimen was administered to 2 patients, all as adjuvant chemotherapy. Most patients with lymph-node metastasis were given adjuvant chemotherapy (Table 2). No patients received radiation therapy.

**Table 1:** Patients’ characteristics

Variable	Patients (*n* = 79)
Age	
Median (range)	68 (42–83)
Sex	
Male	44 (56%)
Female	35 (44%)
Type of pancreatectomy	
PD	45 (57%)
PpPD	31 (39%)
SSPPD	3 (4%)
Node dissection	
D1	24 (30%)
D2	52 (66%)
D3	3 (4%)
Operative time (min)	
Median (range)	466 (293–856)
Blood loss (ml)	
Median (range)	850 (100–3696)
Transfusion	
Yes	19 (24%)
No	60 (76%)
Morbidity	
Clavien-Dindo grade IIIa	35 (44%)
Pancreatic fistula Grade B, C	20 (25%)
Tumor diameter (mm)	
Median (range)	20 (8–65)
Tumor ulceration	
Yes	22 (28%)
No	57 (72%)
Type of adenocarcinoma	
Poorly differentiated	11 (14%)
Moderately differentiated	22 (28%)
Well differentiated	33 (42%)
Papillary	12 (15%)
Mucinous	1 (1%)
T stage (AJCC 8th)	
T1a	21 (27%)
T1b	16 (20%)
T2	15 (19%)
T3a	27 (34%)
N stage (AJCC 8th)	
N0	53 (67%)
N1	22 (28%)
N2	4 (5%)
Overall stage (AJCC 8th)	
IA	20 (25%)
IB	18 (23%)
IIA	15 (19%)
IIIA	22 (28%)
IIIB	4 (5%)
HMGB1 staining intensity	
1 (no staining)	14 (18%)
2 (light brown)	34 (43%)
3 (moderate brown)	23 (29%)
4 (brown or dark brown)	8 (10%)
HMGB1 positive cells (%)	
1 (0–25%)	32 (41%)
2 (26–50%)	13 (16%)
3 (51–75%)	10 (13%)
4 (76–100%)	24 (30%)
HMGB1 expression	
High	31 (39%)
Low	48 (61%)
Adjuvant chemotherapy	
Yes	28 (35%)
Gem	19 (24%)
S-1	7 (9%)
Others	5 (6%)
No	51 (65%)

*PD* pancreaticoduodenectomy, *PpPD* pylorus-preserving pancreaticoduodenectomy, *SSPPD* subtotal stomach-preserving pancreaticoduodenectomy, *AJCC* American Joint Committee on Cancer, *HMGB1* High-Mobility Group Box 1, *Gem* gemcitabine

**Table 2:** Relationship between N stage and adjuvant chemotherapy

	Adjuvant chemotherapy (−)	Adjuvant chemotherapy (+)	*P* value
N0	40	13	0.004
N1, N2	11	15	

### Relationship between HMGB1 expression and other clinicopathological variables

Relationships between HMGB1 expression and other clinicopathological variables are shown in Table 3. Patients with high-expression HMGB1 underwent PD more frequently and showed a larger amount of blood loss than patients with low-expression. None of the tumor factors, including tumor diameter, tumor ulceration, histological type of adenocarcinoma, T state, and N stage, significantly correlated with HMGB1 expression level.

**Table 3:** Relationships between HMGB1 expression and clinicopathological factors

Variable	HMGB1 high (*n* = 31)	HMGB1 low (*n* = 48)	*P* value
Age			
Median (range)	68 (46–83)	66 (42–83)	0.42
Sex			
Male	11 (35%)	24 (50%)	0.21
Female	20 (65%)	24 (50%)	
Type of pancreatectomy			
PD	23 (74%)	22 (46%)	0.013
PpPD, SSPPD	8 (26%)	26 (54%)	
Node dissection			
D1	12 (39%)	12 (25%)	0.20
D2, D3	19 (61%)	36 (75%)	
Operative time (min)			
Median (range)	490 (308–716)	451 (0–856)	0.3
Blood loss (ml)			
Median (range)	949 (150–3696)	722 (100–3020)	0.025
Transfusion			
Yes	8 (26%)	11 (23%)	0.77
No	23 (74%)	37 (77%)	
Morbidity (grade IIIa)			
Yes	13 (42%)	22 (46%)	0.73
No	18 (58%)	26 (54%)	
Tumor diameter (mm)			
Median (range)	20 (8–35)	20 (8–65)	0.074
Tumor ulceration			
Yes	9 (29%)	13 (27%)	0.85
No	22 (71%)	35 (73%)	
Type of adenocarcinoma			
Moderate or poor differentiated	12 (39%)	21 (44%)	0.66
Other types	19 (61%)	27 (56%)	
T stage (AJCC 8th)			
T3a	9 (29%)	18 (38%)	0.44
T1a, T1b, T2	22 (71%)	30 (62%)	
N stage (AJCC 8th)			
N1, N2	12 (39%)	14 (29%)	0.38
N0	19 (61%)	34 (71%)	
Overall stage (AJCC 8th)			
IIA, IIIA, IIIB	17 (55%)	24 (50%)	0.67
IA, IB	14 (45%)	24 (50%)	
Adjuvant chemotherapy			
(−)	20 (65%)	31 (65%)	1.00
(+)	11 (35%)	17 (35%)	

### Prognostic factors for patients with adenocarcinoma of the ampulla of Vater treated with R0 resection

**Fig. 2:**
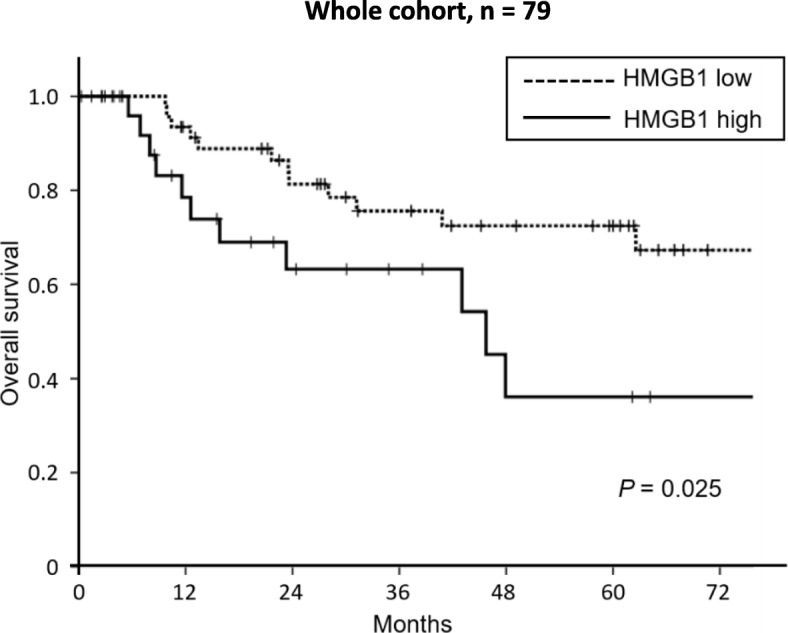
Correlation between HMGB1 expression and survival of patients with adenocarcinoma of ampulla of Vater. High HMGB1 expression is significantly correlated with worse overall survival for patients with adenocarcinoma of the ampulla of Vater as shown with Kaplan-Meier survival curves

The 5-year overall survival rate and median survival time in the whole cohort were 58.1% and 113 months, respectively. Univariate analysis was performed to identify factors affecting overall survival (Table 4). Blood loss (≥ 800 ml), intraoperative transfusion, advanced T stage (T2, T3a), lymph-node metastasis, and HMGB1 high expression were significantly associated with shorter overall survival. Multivariate analysis showed that intraoperative transfusion (hazard ratio [HR] 3.13; 95% confidence interval [CI] 1.26–7.78; *P* = 0.014), lymph-node metastasis (HR 7.43; 95% CI 2.83–19.54; *P* < 0.001), and HMGB1 high expression (HR 3.54; 95% CI 1.37–9.16; *P* = 0.009) were predictors of poor overall survival (Table 4). Kaplan-Meier analysis showed that the 5-year overall survival was significantly less in patients with high HMGB1 expression than in those with low HMGB1 expression (36.1% and 72.4%, respectively; *P* = 0.025; Fig. 2).

**Table 4:** Univariate and multivariate analyses of predictors of overall survival

Univariate analysis				Multivariate analysis		
Variable	HR	95% CI	*P* value	HR	95% CI	*P* value
Age (60 ≤)	0.62	0.26–1.50	0.29	–	–	–
Sex (male)	1.40	0.61–3.22	0.42	–	–	–
Type of pancreatectomy (PD)	1.43	0.63–3.22	0.39	–	–	–
Node dissection (D1)	1.87	0.83–4.23	0.19	–	–	–
Operative time (480 min ≤)	0.97	0.42–2.20	0.93	–	–	–
Blood loss (800 ml ≤)	3.46	1.28–9.34	0.014	1.88	0.60–5.91	0.28
Transfusion (yes)	3.21	1.44–7.17	0.004	3.13	1.26–7.78	0.014
Morbidity (grade IIIa)	0.98	0.44–2.19	0.96	–	–	–
Tumor diameter (20 mm ≤)	1.12	0.47–2.65	0.80	–	–	–
Histological type (moderate or poor)	1.93	0.86–4.32	0.11	–	–	–
T stage (T2, T3a)	2.57	1.05–6.25	0.038	1.31	0.47–3.63	0.60
N stage (N1, N2)	9.12	3.53–23.57	< 0.001	7.43	2.83–19.54	< 0.001
HMGB1 expression (High)	2.48	1.09–5.66	0.030	3.54	1.37–9.16	0.009
Adjuvant chemotherapy (−)	0.70	0.30–1.64	0.42	–	–	–

### HMGB1 expression was significantly prognostic only in patients who did not receive adjuvant chemotherapy

**Fig. 3:**
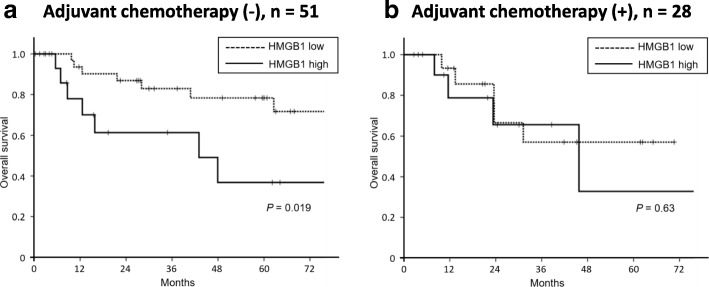
**a**, **b** Lack of correlation between HMGB1 expression and survival of patients with adenocarcinoma of ampulla of Vater treated with adjuvant chemotherapy. High HMGB1 expression was not significantly associated with poorer survival of patients treated with adjuvant chemotherapy

High expression of HMGB1 was a good predictor of survival for patients without adjuvant chemotherapy (5-year overall survival with high and low HMGB1 expression: 36.8% and 71.8%, respectively; *P* = 0.019; Fig. 3a), but not for patients who received adjuvant chemotherapy (5-year overall survival in high and low HMGB1 expression: 32.8% and 57.0%, respectively; *P* = 0.63; Fig. 3b).

## Discussion

The only known treatment to achieve cure for ampullary cancer is surgical resection. Since 4.6 to 9% of T1 ampullary cancer cases have lymph-node metastasis, the standard treatment method in Japan is pancreaticoduodenectomy with lymph-node dissection [2, 4, 11]. It has been shown that T stage, lymph-node metastasis, residual cancer cells, and intraoperative transfusion are predictors of poor prognosis in ampullary cancer [12–16]. Moreover, the pancreatobiliary type on histological subtyping, which was first reported by Kimura et al., has also been demonstrated to be associated with survival [14, 17]. Fluorouracil-based adjuvant chemoradiotherapy may prolong survival of ampullary cancer patients following pancreaticoduodenectomy, but the evidence is limited [18].

HMGB1, a non-histone DNA-binding protein first reported in the calf thymus in 1973, is present in most mammalian cells [19, 20]. Wang et al. showed that HMGB1 was involved in inflammatory responses [21]. Intranuclear HMGB1 participates in several DNA functions, including DNA chaperone, DNA repair, transcription, maintenance of telomeres, and genome stability. Moreover, previous studies reported that HMGB1 enhances the functions of key immune suppressor cells such as myeloid-derived suppressor cells and regulatory T cells, inhibiting anti-tumor immune responses [22, 23]. Furthermore, HMGB1 expression was inversely associated with CD45RO^+^ T cell infiltration in colon cancer [24]. Thus, HMGB1 affects anti-tumor immune responses, but further investigations are necessary to better understand the role of HMGB1 in anti-tumor immunity.

Importantly, recent studies have found that HMGB1 expression is associated with tumorigenesis and tumor progression [25, 26]. HMGB1 appears to play a particular role in apoptosis inhibition, cell proliferation, angiogenesis, invasion, and metastasis in many malignancies [26]. Wu et al. reviewed the impact of HMGB1 overexpression on patient survival in several cancers [5]. Overexpression of HMGB1 was significantly correlated with poorer overall survival in esophageal cancer, gastric cancer, colorectal cancer, pancreatic cancer, hepatocellular carcinoma, nasopharyngeal cancer, head and neck cancer, bladder cancer, cervical cancer, and pleural mesothelioma. Moreover, there are some reports of HMGB1 in biliary tract cancers. RAGE, a receptor binding to HMGB1, was reportedly associated with invasion capacity in a study using bile duct-cancer cell lines [27]. Xu et al. investigated HMGB1 expression in intrahepatic cholangiocarcinoma, concluding that HMGB1 overexpression was significantly correlated with worse survival [28]. The level of HMGB1 expression was reported to be associated with lymph-node metastasis and advanced TNM stage in esophageal cancer, gastric cancer, colorectal cancer, head and neck cancer, and cervical cancer [29–33]. Moreover, HMGB1 expression was positively correlated with lymphatic-vessel density in esophageal cancer and intrahepatic cholangiocarcinoma [28, 29]. These results suggest possible reasons why HMGB1 overexpression may contribute to poorer patient survival.

The present study is the first to examine the association of HMGB1 expression and ampullary cancer. Our study demonstrated that high HMGB1 expression was significantly associated with shorter survival for patients with adenocarcinoma of the ampulla of Vater following R0 resection. HMGB1 was predictive for patients only without adjuvant chemotherapy. Adjuvant chemotherapy for patients with high HMGB1 expression, as well as patients with lymph-node metastasis, may be effective for some patients. Recently, there have been reports on novel HMGB1-targeted therapy in preclinical models. In a mouse model, anti-HMGB1 antibody therapy was reported to reduce colorectal cancer liver metastasis derived from a cell line expressing the highest level of HMGB1 [34]. Similarly, a study using mouse models showed that HMGB1-RAGE-signaling blockade suppressed tumor growth in which both RAGE and HMGB1 were expressed [35]. Based on these results, HMGB1-targeted therapy for ampullary cancer with HMGB1 overexpression is promising. Our results suggest a general phenomenon that has to be further studied.

There are several limitations in this study. First, the total number of patients was not high, despite it being a multi-institutional study. Second, the patients underwent different adjuvant chemotherapy regimens. Finally, the detailed molecular biological mechanisms of HMGB1 examined in the present study were not studied. Even after taking these limitations into consideration, however, the results of the present study suggests a new biomarker for survival, as well as a potential treatment target for adenocarcinoma of the ampulla of Vater. We have not studied the expression of HMGB1 as a function of the histologic subtype and plan to do in future studies.

## Conclusions

The present study suggests that HMGB1 is a prognostic factor and potential biomarker for survival in patients not treated with adjuvant chemotherapy with adenocarcinoma of the ampulla of Vater not treated with adjuvant chemotherapy.

## Data Availability

The datasets used and/or analyzed during the current study are available from the corresponding author on reasonable request.

## Abbreviations

HMGB1: High-Mobility Group Box 1
PD: Pancreaticoduodenectomy
PpPD: Pylorus-preserving pancreaticoduodenectomy
SSPPD: Subtotal stomach-preserving pancreaticoduodenectomy
YCOG: Yokohama Clinical Oncology Group
